# Utilizing patient data: A tutorial on predicting second cancer with machine learning models

**DOI:** 10.1002/cam4.70231

**Published:** 2024-09-20

**Authors:** Hossein Sadeghi, Fatemeh Seif, Erfan Hatamabadi Farahani, Soraya Khanmohammadi, Shahla Nahidinezhad

**Affiliations:** ^1^ Department of Physics, Faculty of Sciences Arak University Arak Iran; ^2^ Department of Radiotherapy and Medical Physics Arak University of Medical Sciences & Khansari Hospital Arak Iran; ^3^ Industrial and Systems Engineering, Tarbiat Modares University Tehran Iran

**Keywords:** decision trees, machine learning, precision medicine, radiation dosage

## Abstract

**Background:**

The article explores the potential risk of secondary cancer (SC) due to radiation therapy (RT) and highlights the necessity for new modeling techniques to mitigate this risk.

**Methods:**

By employing machine learning (ML) models, specifically decision trees, in the research process, a practical framework is established for forecasting the occurrence of SC using patient data.

**Results & Discussion:**

This framework aids in categorizing patients into high‐risk or low‐risk groups, thereby enabling personalized treatment plans and interventions. The paper also underscores the many factors that contribute to the likelihood of SC, such as radiation dosage, patient age, and genetic predisposition, while emphasizing the limitations of current models in encompassing all relevant parameters. These limitations arise from the non‐linear dependencies between variables and the failure to consider factors such as genetics, hormones, lifestyle, radiation from secondary particles, and imaging dosage. To instruct and assess ML models for predicting the occurrence of SC based on patient data, the paper utilizes a dataset consisting of instances and attributes.

**Conclusion:**

The practical implications of this research lie in enhancing our understanding and prediction of SC following RT, facilitating personalized treatment approaches, and establishing a framework for leveraging patient data within the realm of ML models.

## 1 INTRODUCTION

The occurrence of secondary cancer (SC) following RT is a significant concern and a potential long‐term complication of this treatment. It is crucial to minimize the risk of developing SC, considering that the main objective of RT is to provide effective treatment to patients. Several factors contribute to the likelihood of SC, including the radiation dose and volume received by the patient, the patient's age at the radiotherapy, the specific organ or tissue being irradiated, and genetic predisposition. To reduce this risk, it is necessary to establish new modeling techniques that incorporate semi‐empirical relationships.^1^, ^2^, ^3^

Since the early stages of the development of RT and its implementation, the potential risk of SC due to radiation absorption by healthy tissues has been a concern. In 1948, one of the initial mathematical models was introduced to assess the probability of SC because of RT.^4^ As the field of RT advanced, so did the modeling and understanding of the risk of SC.^5^ The newer models incorporated the presence of precancerous cells. Initially, the relationships between dose absorption and SC were primarily considered, but it was later recognized that various factors, such as the patient's age at the radiotherapy and the duration of follow‐up, also played a role in the probability of SC. In 2009, these factors were revised and considered.^6^ Despite significant progress in mathematical and theoretical modeling in recent years, no single reference model has been able to encompass all the parameters because of the non‐linear characteristics of their dependencies. Furthermore, the existing models did not consider the influence of factors such as genetics, hormones, lifestyle, radiation from secondary particles in RT, and the dose received from imaging.^7^ Numerous practical studies have been conducted to align theoretical models with experimental data, resulting in the determination of SC risk for numerous patients. The MCNPX code has served as a valuable tool in facilitating this comparison between modeling and experimental data.^8^

ML has been implemented in the medical domain to aid in case‐based reasoning and enhance the precision of diagnoses and prognostic decisions.^9^, ^10^ In the realm of BC, ML techniques have been utilized to anticipate and categorize cases as either benign or malignant.^11^, ^12^, ^13^, ^14^ Moreover, these techniques have been employed to forecast the recurrence of cancer by analyzing clinical data, genetic data, and medical images.^15^, ^16^ Nevertheless, it is crucial to acknowledge the dearth of studies that have employed ML to predict the incidence of second primary breast cancer (SPBC) or a combination of recurrent and SPBC.^17^, ^18^, ^19^, ^20^ Recently, a feed‐forward neural network has been devised as an automated tool to aid clinicians in identifying women who have a high risk of SPBC, potentially enabling preventive measures. This signifies a noteworthy advancement in the field and underscores the potential of ML in enhancing BC care.^21^

The purpose of this research was to examine different models to determine the best ML model to determine the basic features that can be easily available to predict the occurrence of SC based on patient data. In addition, the aim was to categorize patients into high‐risk or low‐risk groups for developing an SC.

The document is structured in the following manner: Section 2 presents a comprehensive overview of the various ML models examined to determine the best ML model for predicting the occurrence of SC based on patient data. Section 3 provides a comparison of the results obtained from the ML models and discusses the preferred method for determining feature importance and the prediction of SC risk. Finally, Section 4 delivers a succinct summary of the findings and draws reasoned conclusions from the conducted research.

## 2 ML MODELS AND METHODOLOGY

**FIGURE 1:**
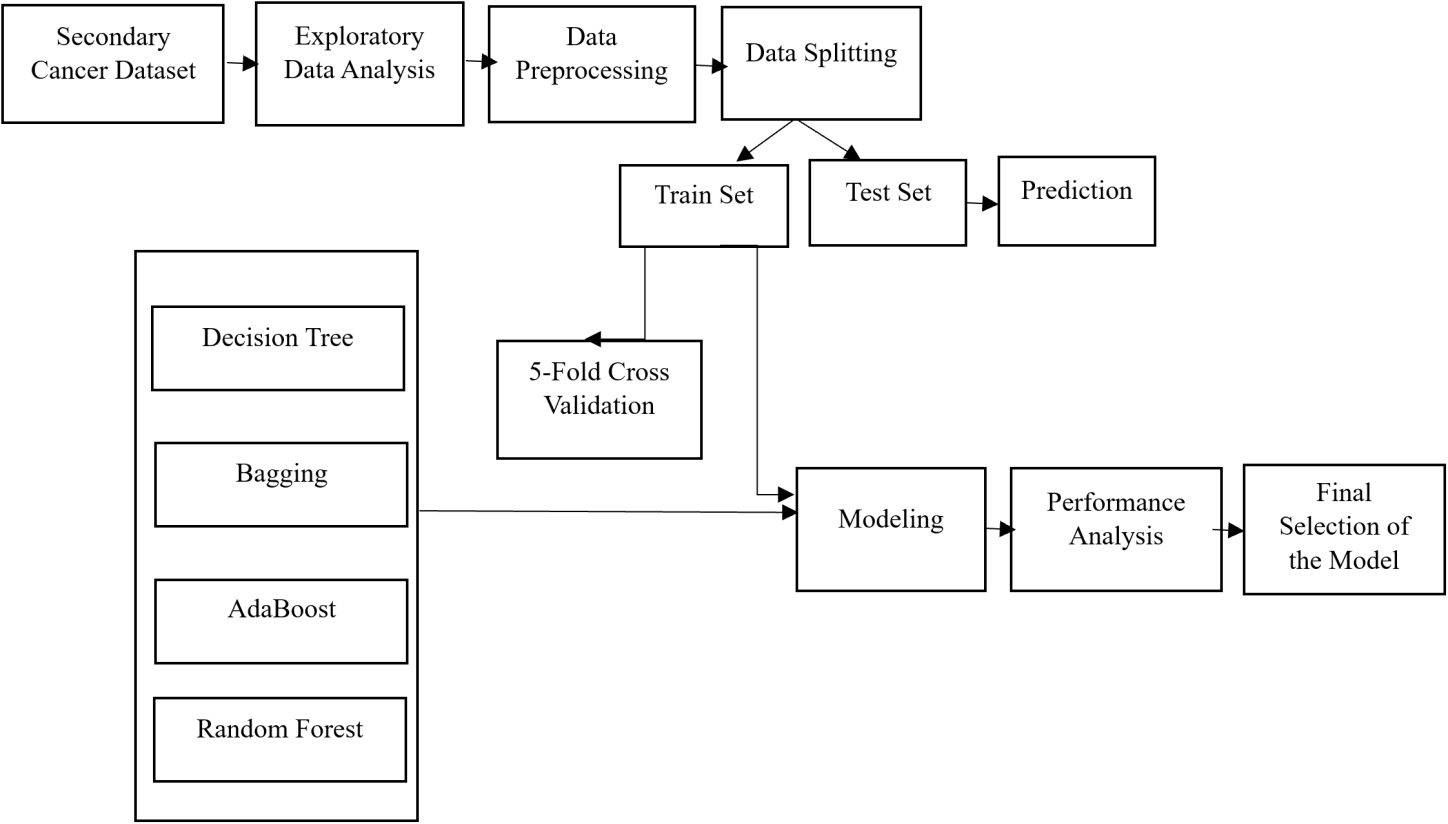
Overall workflow diagram.

There is a visual representation of the research process, which is depicted in Figure 1. This figure serves as a workflow diagram, illustrating the overall process of conducting research. Initially, data collection is done to gather and record information from various sources. Next, the dataset undergoes preprocessing. The following phase involves creating a prediction model of SC using the Decision Tree (DT) algorithm and three ensemble learning algorithms known as Random Forest (RF), Bagging, and AdaBoost. In the next step, we will determine the optimal hyperparameters and assess the generalizability and robustness of the models. Finally, common evaluation metrics are used to assess the effectiveness of the proposed models.

### 2.1 Data collection and study populations

An analysis was conducted on 21 experimental and computational studies involving patients who underwent radiotherapy. The dataset consists of 65 studies conducted between 1980 and 2000, each focusing on different types of SCs. Within these 65 studies, there were 23 distinct types of SCs examined. The number of studies investigating each specific type of SC varied, with 16 studies dedicated to SBC and 5 studies dedicated to stomach cancer, for instance. Ultimately, we have gathered all available data from a total of 113 studies, which provide valuable information regarding the number of SC cases or mortality, the percentage of women included in the studies, the period during which individuals were subjected to radiation, the age at which follow‐up was conducted, and the average radiation dose administered. The dataset holds the utmost importance for any ML model. In this study, we employed a dataset comprising 113 instances and 8 attributes to train and test our models. Within this dataset, there are 67 samples classified under the "incidence" class and 46 samples classified under the “mortality” class. Table 1 provides a concise summary of a brief dataset's attributes and features. The first column of Table 1 lists the publication years of each study, while additional details, such as follow‐up, age at exposure, dose range and its average, and cancer site are provided in the subsequent columns. Among the studies reviewed, the majority originated from the United States, accounting for eight cases, followed by five studies that utilized population‐based data from various countries. Sweden contributed three studies, Israel provided two, and the Netherlands, England, and Scotland each had one study represented. The research methodologies varied, with one study employing a case‐control design, three adopting a cohort approach, and the remaining 17 studies (approximately 81%) classified as population‐based. In terms of data sources, most studies were derived from hospital records (*n* = 10), followed by cancer registry data (*n* = 6), and university research (*n* = 5). The cumulative participant count across all studies reached 371,992, with a mean of 17,714 and a range from 601 to 182,040.

**TABLE 1:** Dataset's characteristics of SC in test samples.

A summary of the dataset's characteristics								
Publication	All case	Cases/death	% Women in the study	age at exposure	Follow‐up (years)	Average dose (Sv)	Dose Range (Sv)	Cancer site
Weiss et al. (1994)^22^	14109	148	17.8	<25 to >55	5 to >35	5.55	0 to >10.16	Esophagus
Griem et al. (1994)^23^	3609	61	21.2	<35 to >55	1 to 51	14.8	0 to 14.8	Stomach
Inskip et al. (1990)^24^	4153	73	100	13 to 88	0 to 60	1.2	<0.6 to 6.65	Colon
Darby et al. (1995)^25^	2067	47	100	23 to 65	5 to 49	3.2	<2.41 to >3.73	Colon
Weiss et al. (1994)^22^	14109	226	17.8	<25 to >55	5 to >35	4.1	0 to >7.85	Colon
Darby et al. (1995)^25^	2067	14	100	23 to 65	5 to 49	4.9	<3.32 to >6.51	Rectum
Mattsson et al. (1997)^26^	1216	31	100	8 to 74	5 to 61	0.66	0 to 5.39	Liver
Inskip et al. (1990)^24^	4153	35	100	13 to 88	0 to 60	0.16	<0.08 to 0.87	Pancreas
Griem et al. (1994)^23^	3609	9	21.2	<35 to >55	1 to 51	0.08	0 to 0.08	Larynx
Kaldor et al. (1992)^27^	25665	40	15.3	52 to 45	1 to >10	Unknown	0 to >3.9	Lung
Van Leeuwen et al. (1995)^28^	1939	30	41	<45 to >55	1 to >10	7.2	0 to >21	Lung
Mattsson et al. (1997)^26^	1216	19	100	8 to 74	5 to 61	0.75	0 to 8.98	Lung
Davis et al. (1989)^29^	13385	69	48.7	<24 to >38	0 to 50	0.84	0 to >8	Lung
Boice et al. (1988)^30^	182040	15	100	<30 to >75	1 to >40	22	0 to >32	Bone
Shore et al. (1984)^31^	1680	41	15	1 to 19	10 to 49	5	0 to 5.5	NMSC
Hildreth et al. (1985)^32^	2650	24	42	<1	5 to >48	2.25	0 to >2.25	NMSC
Shore et al. (1986)^33^	601	14	100	<19 to >40	0 to 45	2.6	0 to >2.6	NMSC
Boice et al. (1989)^34^	12040	140	100	<30 to >75	5 to >40	0.31	0 to 0.98	Breast
Hildreth et al. (1989)^35^	1201	34	100	<1	0 to >52	0.69	0.01 to 7.1	Breast
Weiss et al. (1994)^22^	14109	176	17.8	<25 to >55	5 to >35	2.18	0 to >4.85	Prostate
Griem et al. (1994)^23^	3609	15	21.2	<35 to >55	1 to 51	Unknown	0 to 0.17	Bladder
Weiss et al. (1994)^22^	14109	142	17.8	<25 to >55	5 to >35	2.18	0 to >4.85	Bladder
Boice et al. (1988)^30^	182040	43	100	29 to 8	5 to >40	0.11	0.01 to 0.24	Thyroid
Ron et al. (1989)^36^	10834	60	51	0 to 15	<15 to >30	0.09	0.04 to 0.5	Thyroid
Pottern et al. (1990)^37^	602	81	38.7	0.4 to 16	0 to 46	0.24	0 to 0.53	Thyroid
Hall et al. (1996)^38^	23319	50	78	1 to 75	5 to 39	0.8	0 to 29.2	Thyroid
Damber et al. (1995)^39^	20024	81	49.4	<20 to >70	0 to >19.6	0.39	<0.06 to >1.04	NHL
Ron et al. (1988)^40^	10834	16	51	0 to 15	0 to >28	0.3	0 to >0.3	NHL
Griem et al. (1994)^23^	3609	21	21.2	<35 to >55	1 to 51	1.55	0 to 1.55	NHL
Damber et al. (1995)^39^	20024	17	49.4	<20 to >70	0 to >19.6	0.39	<0.06 to >1.04	Hodgkins
Damber et al. (1995)^39^	20024	65	49.4	<20 to >70	0 to >19.6	0.39	<0.06 to >1.04	Multiple myeloma
Kaldor et al. (1990)^41^	29552	163	35	42 to 37	1 to >10	Unknown	0 to >20	Leukemia
Boivin et al. (1995)^42^	10472	122	42.4	<15 to >55	1 to 44	Unknown	0 to >30	Leukemia
Damber et al. (1995)^39^	20024	61	49.4	<20 to >70	0 to >19.6	0.39	<0.06 to >1.04	Leukemia

The collection of data that encompasses the association between SC risk and radiation dose, as well as factors such as sex and age of radiation exposure is crucial for our research. The likelihood of health effects resulting from radiation exposure is influenced by factors such as the age at which exposure occurs, the sex of the individual, the specific calendar year, tissue types, and the attained age of SC. Research conducted on underground miners indicates that a younger age at the time of exposure correlates with an increased risk of developing lung cancer.^43^ Likewise, among survivors of childhood cancer, the incidence of primary hypothyroidism following radiation treatment is notably higher in females and in those who were exposed after the age of 15.^44^ Additionally, the cumulative dose of red bone marrow from diagnostic radiation is affected by the calendar year, exhibiting peaks around 1950 and post‐1980, with men generally receiving higher doses than women.^45^ These observations highlight the necessity of accounting for age, sex, and temporal variables when evaluating radiation‐related health risks in epidemiological research. To obtain this information about a specific group of patients, we have relied on studies conducted in the past that examined exposure to radiation during medical treatments. The studies presented in Table 1 were compiled and concluded by various groups over the past few decades. The Transparent Reporting of a Multivariable Prediction Model for Individual Prognosis Or Diagnosis (TRIPOD) offers a comprehensive checklist of items designed to ensure transparent reporting in studies involving prediction models. This initiative seeks to improve the evaluation of potential biases and the overall utility of these models.^46^ Considering the growing integration of artificial intelligence within prediction models, an extension known as TRIPOD‐AI, along with a related risk‐of‐bias assessment tool called PROBAST‐AI, is currently under development.^47^ These instruments are intended to enhance the reporting standards and critical evaluation of studies that utilize ML for prediction, thereby aiming to minimize research waste and improve the assessment of both study quality and outcomes. For example, we are utilizing the data collected by little to employ artificial intelligence and ML methods to predict the risk of SC.

### 2.2 Data preprocessing

Data preparation is a crucial step in developing predictive models. It involves addressing common issues such as handling missing values, infinite values, and rare categorical levels. The process typically includes four main steps: data acquisition, cleaning, preprocessing, and ensuring consistency. Data preprocessing remains an important step in ML methods. The appropriate preprocessing of imbalanced data is crucial, as it allows researchers to minimize defects to the greatest extent possible, potentially resulting in the complete eradication of defects within current data sets. Comprehensive literature reviews have underscored the significance of data preprocessing in ML research.^48^ In Table 2, a brief synopsis of the dataset's characteristics and the statistical properties of the dataset's features are presented, with a focus on the minimum, maximum, mean, standard deviation, and other features. Exploratory data analysis (EDA) is employed to thoroughly analyze and investigate the dataset, and summarize its main characteristics.

**TABLE 2:** Statistical description of the numerical features.

Feature	Type	Min	Max	Mean *±* SD	Description
Cancer site	Nominal				Location of the cancer
All case	Numeric	257	182,040	28310.79 *±* 2233.03	The overall count of individuals involved in the research.
Cases/death	Numeric	6	1178	108.12 *±* 180.71	The number of individuals in two modes: “**incidence**” and “**mortality**”
%women in the study	Numeric	0	100	61.32 *±* 33.52	The percentage of women participants
The average age at exposure	Numeric	0	75	34.28 *±* 18.88	The average age at which individuals are first exposed to cancer
Average follow‐up age	Numeric	4.9	45	24.03 *±* 9.02	The average age at which individuals start referring for cancer screening tests
Average dose (Sv)	Numeric	0.01	165	7.52 *±* 17.42	The received average dose in Sv

**FIGURE 2:**
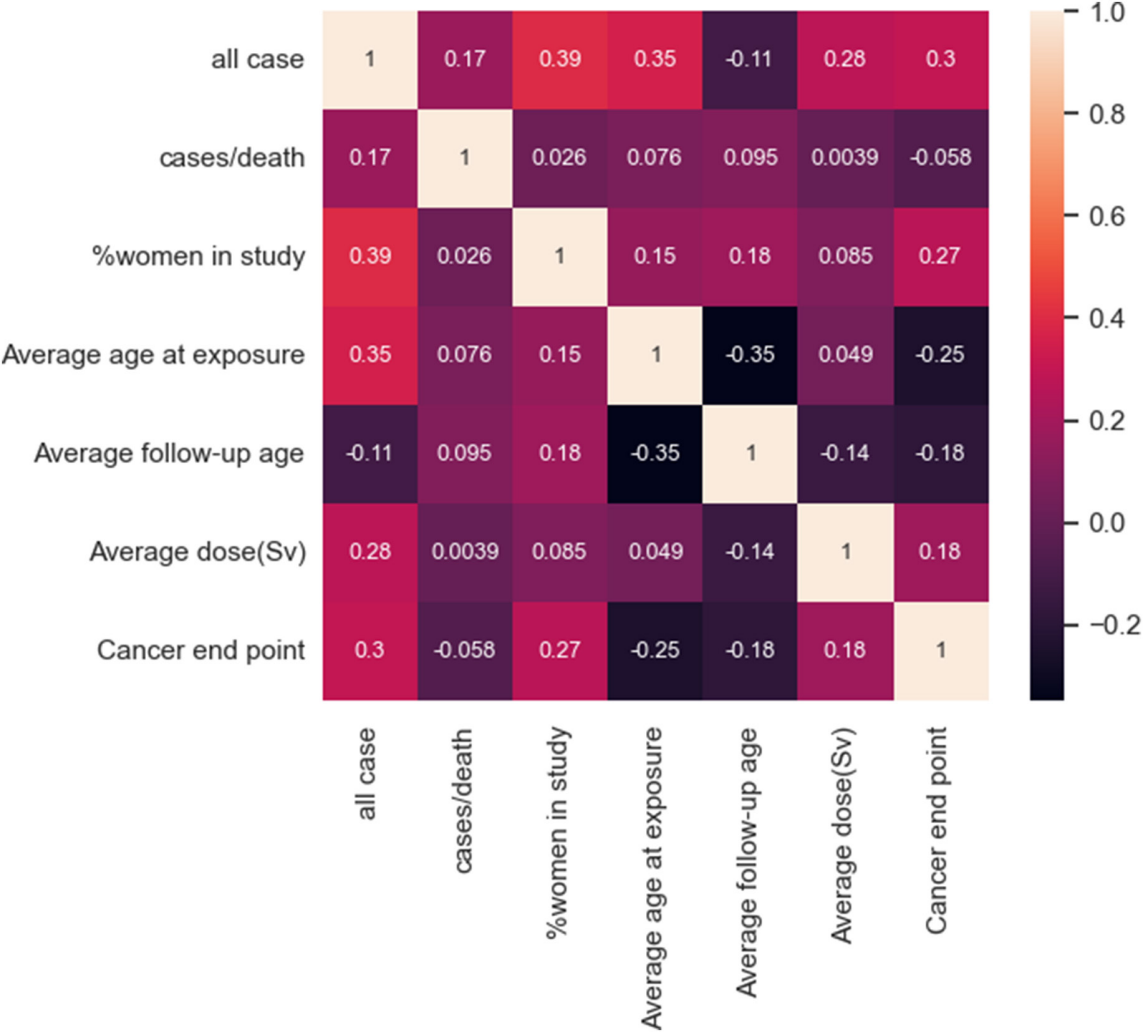
Correlation heat map plot.

Figure 2 correlation heat map, which provides insight into the relationship between variables. The heat map reveals a little correlation between the target variable and the features. Data preprocessing is an essential component in the data mining process as it significantly impacts the predictive model performance. The step encompasses cleaning, transforming, and integrating the data to prepare it for analysis. In this particular study, some missing values were replaced with the mean and others were excluded from the study. When variables are assessed using distinct scales, their contributions to the model fitting are not equal and can lead to bias. To address this issue, normalization is employed before model fitting. There are various well‐known normalization methods available, including Simple Feature Scaling, Min‐Max, and *Z*‐score.^49^ In this paper, the Min‐Max normalization method is utilized. The Min‐Max scaling equation is as follows:

(1) $$X_{\mathrm{norm}}=\frac{X_{\mathrm{old}}-X_{\min}}{X_{\max}-X_{\min}}$$

### 2.3 Feature extraction

In our analysis, we utilized the original features present in the dataset without performing any specific feature extraction techniques. The focus of my work is on applying various machine learning algorithms to improve the model's performance. We used the features as they were provided in the dataset, directly employing them in the modeling process without any additional extraction or transformation (Table 3).

**TABLE 3:** Stratified fivefold cross‐validation.

5‐Fold	Model	Accuracy	Precision	Recall	*F*‐score
DT					
Fold‐1		0.87	0.80	1.00	0.84
Fold‐2		0.81	0.87	0.77	0.82
Fold‐3		0.75	0.80	0.90	0.80
Fold‐4		0.62	0.72	0.90	0.70
Fold‐5		0.66	0.66	0.77	0.76
5‐Fold mean		0.75	0.77	0.87	0.79
5‐Fold SD		0.09	0.07	0.08	0.05
Bagging					
Fold‐1		0.81	0.80	0.88	0.84
Fold‐2		0.87	1.00	0.88	0.88
Fold‐3		0.93	0.88	0.80	0.88
Fold‐4		0.81	0.76	1.00	0.86
Fold‐5		0.73	0.75	0.77	0.76
5‐Fold mean		0.83	0.84	0.87	0.85
5‐Fold SD		0.07	0.09	0.08	0.05
AdaBoost					
Fold‐1		0.87	0.81	1.00	0.90
Fold‐2		0.93	1.00	0.88	0.94
Fold‐3		0.81	1.00	0.70	0.82
Fold‐4		0.81	0.76	1.00	0.86
Fold‐5		0.93	1.00	0.88	0.94
5‐Fold mean		0.87	0.92	0.90	0.90
5‐Fold SD		0.05	0.1	0.11	0.04
RF					
Fold‐1		0.81	0.80	0.88	0.90
Fold‐2		0.93	1.00	0.88	0.94
Fold‐3		0.87	1.00	0.90	0.90
Fold‐4		0.75	0.76	0.90	0.81
Fold‐5		0.93	1.00	0.88	1.00
5‐Fold mean		0.86	0.91	0.89	0.91
5‐Fold SD		0.07	0.11	0.01	0.06

### 2.4 Model selection

The development of intelligent predictive models aimed at health outcomes necessitates meticulous attention to the processes of data collection, preprocessing, and feature selection. Following the completion of data preprocessing, the dataset was partitioned into two distinct subsets: a training set, which accounted for 70% of the data, and a testing set, which comprised the remaining 30%. Additionally, four distinct ML models were thoroughly investigated. We conducted a study on ML models to implement them in the aforementioned dataset. We utilized the DT algorithm and explored Ensemble ML algorithms such as Bagging, AdaBoost, and RF. The DT is known to be an efficient algorithm for classification problems.^50^ Bagging, Boosting, and RF are rare popular ensemble learning techniques that integrate several base learners to form a composite model, thereby enhancing accuracy and reliability in performance.^51^ Ensemble learning is a potent technique that combines several individual classifiers to create a robust classifier. Numerous research efforts have demonstrated that models utilizing ensemble learning exhibit enhanced generalization capabilities and yield superior performance on imbalanced datasets.^52^ Therefore, in our study, we investigated the DT algorithm and three ensemble learning algorithms known as RF, Bagging, and AdaBoost for the classification of secondary cancer. Each model is presented below with a concise description:

- **DT**: DT is a widely used classification algorithm because of its interpretability and simplicity in implementation. This methodology entails the creation of a tree structure that incorporates rules to collectively carry out the classification process. The structure of the tree consists of internal nodes, branches, and leaf nodes, which represent attributes, attribute values, and classes found within the dataset, respectively. An internal node, referred to as a branch, generates an output that acts as input for another internal node.^53^
- **Bagging**: Breiman^54^ introduced the concept of bagging, also referred to as bootstrap aggregation. This widely used ensemble technique generates ultimate predictions by randomly selecting subsets of the data. By employing a randomization approach in prediction generation, this meta‐estimator effectively diminishes the variation it produces. Moreover, it aids in mitigating overfitting in intricate algorithms.^55^
- **AdaBoost**: AdaBoost, an adaptive boosting technique, is a classification algorithm that was proposed by Freund& Schapire,^56^ aimed to enhance the precision of classification‐oriented ML approaches. The initial step in AdaBoost involves the creation of a DT for training purposes, where each data point is assigned an equal weight. Subsequently, the fitted model is utilized to classify the entire training set. The weights of correctly predicted instances remain unchanged, while the weights of misclassified instances are increased. Following the normalization of the weights across all training datasets, a new DT is generated using a randomly sampled subset of the data. This iterative process continues until a specific condition is satisfied. Ultimately, by aggregating the weighted sum of all the DTs, the final DT is constructed.^57^
- **RF**: Conversely, RF stands as an ensemble learning classification algorithm comprising numerous sub‐DTs. These sub‐trees are formed through the utilization of bagging and feature randomness techniques, culminating in an uncorrelated collection of trees that collectively yield superior prediction accuracy in comparison to any single tree.^58^ Ho introduced RF in 1995, drawing inspiration from the random subspace method, to construct a diverse array of DTs that effectively mitigate overfitting concerns during the training process.^57^

### 2.5 Grid search and cross‐validation

In this work, the Grid Search approach is applied for tuning classifiers and attempting to identify the best hyperparameters. After tuning the hyperparameters with the grid search method, cross‐validation was used to prevent data leakage and help to reduce variance. The application of stratified fivefold cross‐validation in the imbalanced dataset allows for the mitigation of overfitting. Testing the model on different subsets of the data ensures that the model's performance is not limited to one specific split but can be generalized well across various samples. This approach effectively measures the efficiency of the models in handling the imbalanced dataset. The high average scores from cross‐validation indicate that the proposed prediction model of SC for secondary cancer is likely to generalize well to unseen data and maintain consistent performance. This means that the proposed prediction model of SC is performing effectively across various subsets of the dataset. The proposed prediction models of SC with strong generalization ability refer to their capability to perform well on unseen data, not just the data they were trained on. This means they can make accurate predictions on new and real‐world data.

### 2.6 Performance evaluations

The evaluation of the ML models was conducted through a range of performance metrics. To determine the effectiveness of the models, several performance indicators were employed, including accuracy, precision, recall, F1‐score, and the area under the receiver operating characteristic curve (ROC‐AUC). The mathematical expressions for these metrics are given, where TP, TN, FP, and FN represent true positive, true negative, false positive, and false negative, respectively. To assess the effectiveness of a classification task, it is essential to employ a variety of metrics. Accuracy, in particular, quantifies the ratio of correctly predicted instances to the overall number of instances in the dataset. It is computed by taking the total of TPs and TNs and dividing it by the total count of TPs, FPs, FNs, and TNs.

Precision is another metric that quantifies the proportion of retrieved samples that are relevant. Precision is defined as the ratio of TPs to the total of TPs and FPs. In contrast, recall, which is also referred to as sensitivity or TP rate, measures the effectiveness of a model in correctly identifying positive instances. Recall is calculated by taking the number of true positives and dividing it by the sum of TPs and FNs. The F‐measure, particularly the F1 score, is a metric that integrates both precision and recall into a unified measure. This is achieved by computing the harmonic mean of the two metrics. The F‐measure thus provides a holistic assessment of a model's predictive capabilities, yielding important insights. The confusion matrix for all classifiers is presented in Table 4.

**TABLE 4:** Confusion matrix for all the classifiers.

Confusion matrix classifier	Predicted	
Decision tree		
Actual	0 (mortality) TN	1 (incidence) FP
0 (mortality)	TN = 13	FP = 1
1 (incidence)	FN = 1	TP = 19
Bagging		
Actual	0 (mortality)	1 (incidence)
0 (mortality)	13	1
1 (incidence)	1	19
AdaBoost		
Actual	0 (mortality)	1 (incidence)
0 (mortality)	13	1
1 (incidence)	0	20
RF		
Actual	0 (mortality)	1 (incidence)
0 (mortality)	14	0
1 (incidence)	0	20

**FIGURE 3:**
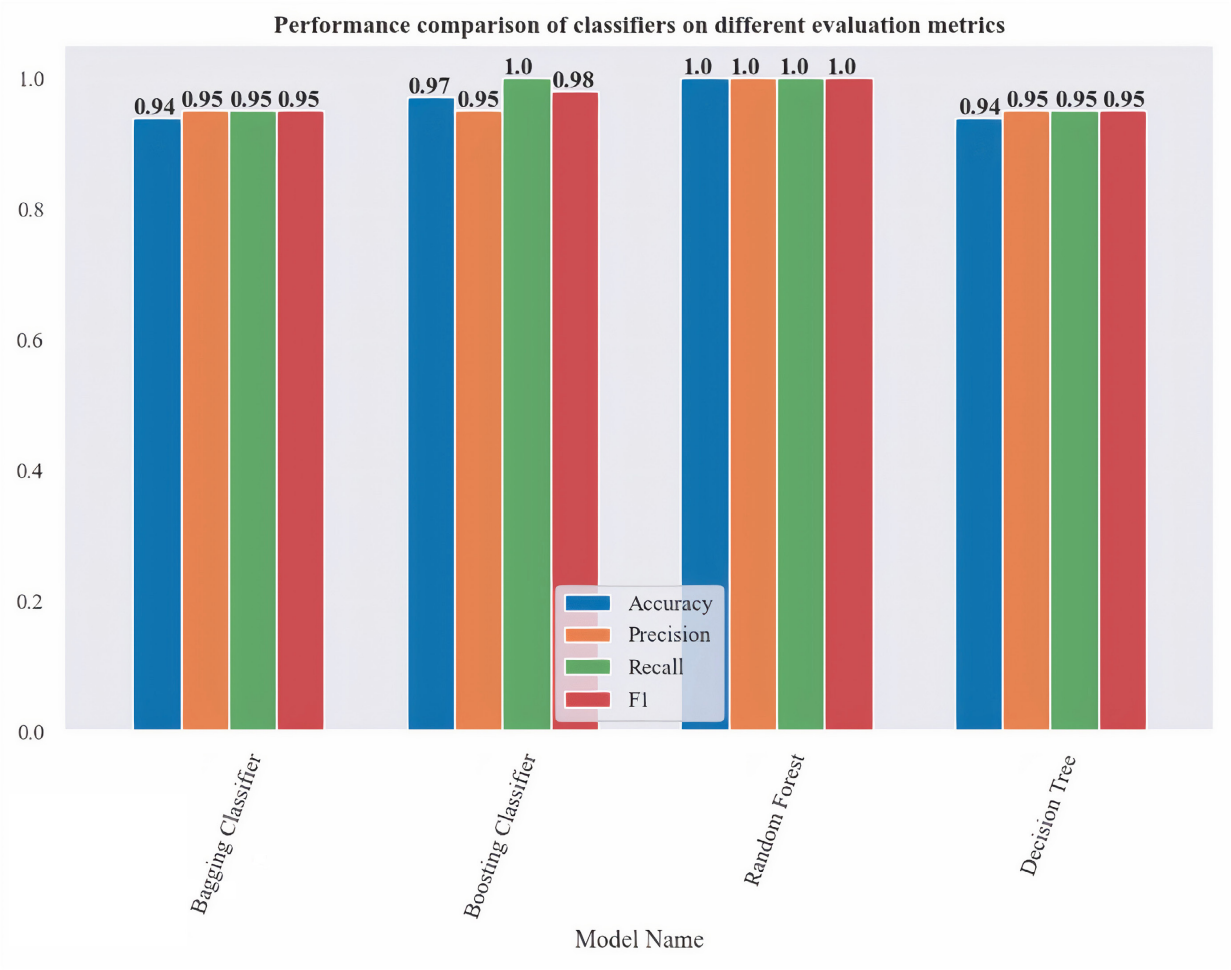
Overall comparative analysis.

Figure 3 illustrates a comprehensive evaluation of the performance of ML models. This evaluation takes into account various well‐known metrics commonly used to assess the performance of prediction models of SC, such as Accuracy, Precision, Recall, and F‐Measure. These metrics provide important information regarding various dimensions of the model's performance. Each classifier is assessed based on F1 score, precision, accuracy, and recall. Accuracy assesses the general precision of the classifier, which is the ratio of correctly predicted instances to the total instances. A perfect accuracy score of 1.0 means that the classifier correctly predicted every instance. Precision indicates how many of the instances predicted as positive are positive. A precision score of 1.0 means that every positive prediction made by the classifier was correct. Recall measures how many of the actual positive instances were correctly identified by the classifier. A recall score of 1.0 means that the classifier identified all positive instances correctly. The F1 Score represents the excellent balance of precision and recall, serving as a metric that balances these two important measures. This is particularly advantageous in scenarios where it is essential to achieve a compromise between precision and recall. A perfect F1 score of 1.0 indicates that the classifier has an excellent balance between precision and recall. Based on the bar chart provided in Figure 3, RF has achieved a score of 1.0 in all four metrics (F1 score, precision, accuracy, and recall). This means it has a perfect performance in identifying both positive and negative instances, making no errors in its predictions. The perfect scores across all metrics indicate that RF is not only accurate but also consistent in its performance. It does not sacrifice one metric for another, which is crucial in many real‐world applications where both precision and recall are important. Therefore, RF is the best choice for classifying secondary cancer, as it has achieved perfect scores of 1.0 across all evaluation metrics: Accuracy, F1 score, precision, accuracy, and recall. With perfect scores, we can trust RF to make accurate predictions. This indicates that RF performs exceptionally well in all aspects of classification, making it the most reliable and effective choice among those presented. Consequently, it can be inferred that the RF classifier outperforms the other classifiers and demonstrates superior performance.

**FIGURE 4:**
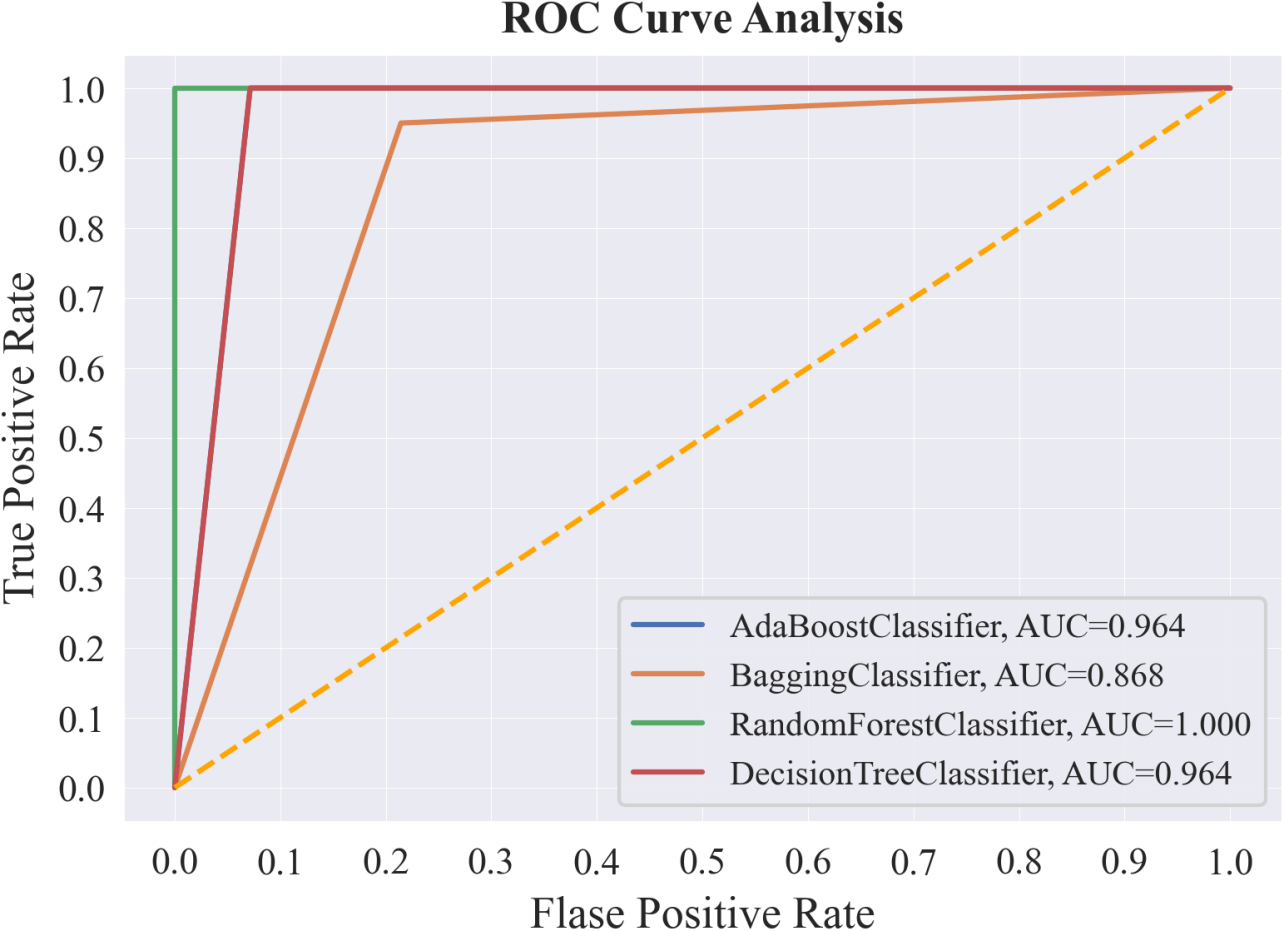
Receiving operator characteristics (ROC) and area under the ROC curve (AUC) for four models.

In addition, to conduct a more thorough examination of each ML model, ROC curves have been generated and are presented in Figure 4. The depicted curves illustrate the performance of the classifiers, highlighting the balance between the TP rate and the FP rate across different classification thresholds. The AUC functions as an indicator of the ML model's capacity to distinguish between different classes, with values spanning from zero to one. A higher AUC value indicates a stronger capability to effectively differentiate the classes.^59^ Typically, the AUC value falls within the range of 0 to 1 and can be categorized as poor (ranging from 0.5 to 0.6), average (from 0.6 to 0.7), satisfactory (spanning 0.7 to 0.8), highly satisfactory (covering 0.8 to 0.9), and excellent (from 0.9 to 1).

Figure 3 displays the ROC curves and AUC values associated with each classifier. Each line on the plot represents a distinct classifier. Notably, the RF classifier exhibits the highest AUC value of 1, that classifier has demonstrated exceptional performance in comparison to the other classifiers. Following the RF classifier, the AdaBoost classifier and the DT classifier both achieve an AUC value of 0.964, while the Bagging classifier lags with an AUC value of 0.868.

**FIGURE 5:**
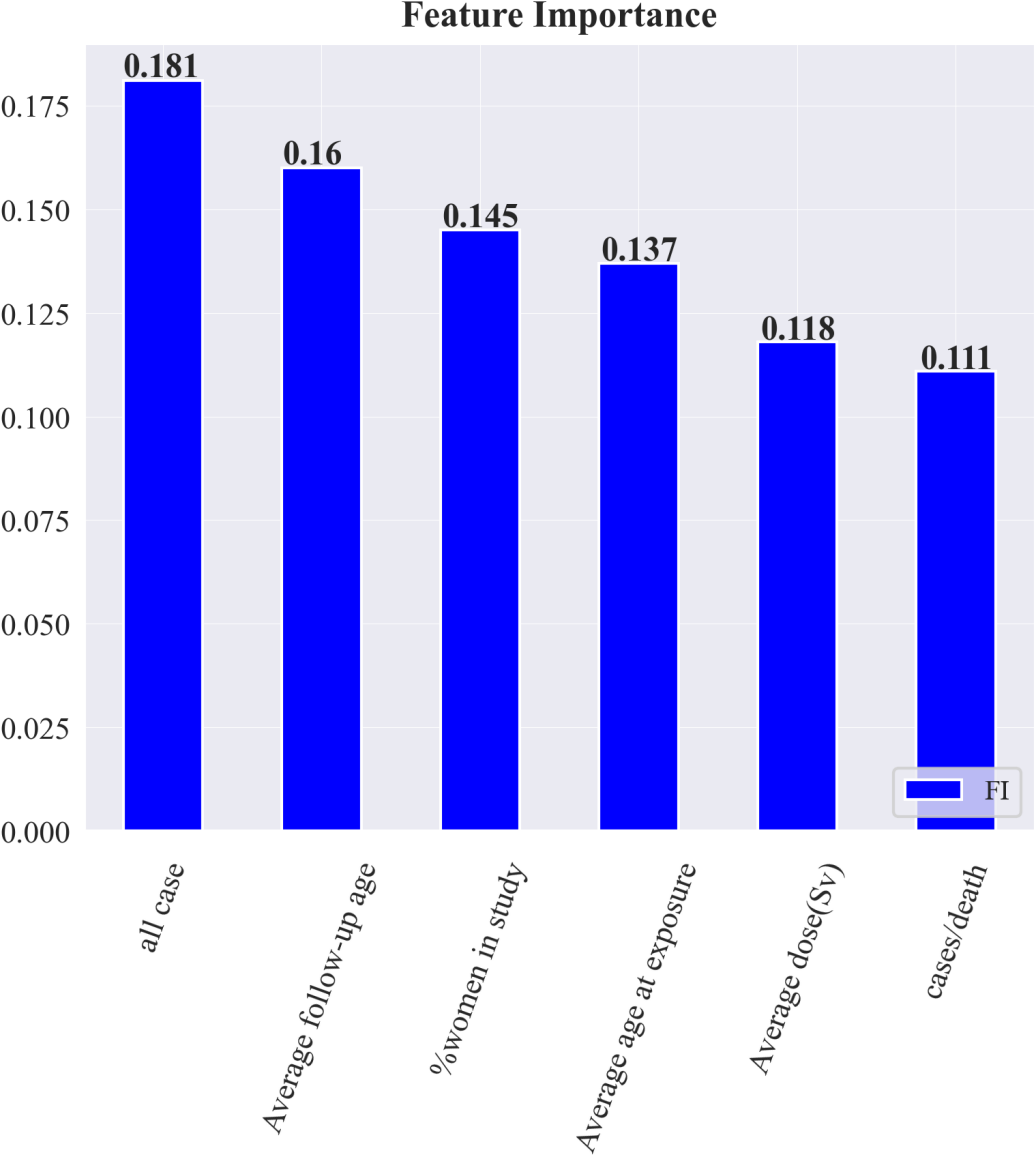
Importance of features.

RF was selected as the preferred method for determining feature importance and the probability of SC in test samples due to its outstanding performance across all evaluation metrics. Assessing feature importance is a valuable technique for interpreting and analyzing the most significant features. The evaluation of feature importance is conducted using the RF approach. The feature importance is shown in Figure 5.

Methods including feature selection, data balancing, and the management of missing values can greatly influence the effectiveness of a model. Research indicates that the interplay among various preprocessing choices can alter predictive results, highlighting the necessity for a more systematic methodology in applied predictive modeling. Investigations emphasize the critical nature of thoughtfully evaluating preprocessing strategies and their possible interactions to enhance both model performance and interpretability.

## 3 RESULTS AND DISCUSSION

In 1995, an investigation was carried out by Neugut et al^61^ to evaluate the potential risk associated with SC in individuals with primary pancreas cancer. Due to the high mortality rate associated with primary pancreas cancer, accurately calculating the risk of SC posed a challenge. To overcome this, the researchers utilized a large population database consisting of nine data centers across the United States (US). The data centers presented a significant portion, approximately 10%, of the total population of the United States. By analyzing the information obtained from these databases, Neugut et al^61^ identified 1.6 million cases of primary cancer that occurred between 1973 and 1990. They then examined the medical records of these patients to identify individuals who subsequently SC. The risk of SC for specific types of cancer was calculated using a confidence interval of 95% confidence interval (95%CI).

However, in 2012, Donovan et al^60^ took a different approach to calculating the risk of SC after radiotherapy. They conducted an experimental study using a phantom and measured the effective dose in the organ using thermoluminescent dosimeters. This allowed them to calculate the risk of SC associated with different radiotherapy techniques, such as whole‐breast radiotherapy (WBRT), accelerated partial breast irradiation (APBI), and simultaneous integrated boost (SIB) with two and three‐volume models. However, in 2012, Donovan et al^60^ took a different approach to calculate the risk of SC after RT. They conducted an experimental study using a phantom and measured the effective dose in the organ using thermoluminescent dosimeters. This allowed them to calculate the risk of SC associated with different radiotherapy techniques, such as WBRT, APBI, and SIB with two and three‐volume models. The key difference between these models lies in the range of radiation doses and their fractionation. For instance, in WBRT, a dose of 40 Sv is delivered in 15 fractions, while APBI involves a dose of 38.5 Sv delivered in 10 fractions.

The range of radiation doses and their fractionation is the primary distinction between these models. To illustrate, WBRT administers a dose of 40 Sv over 15 fractions, whereas APBI entails a dose of 38.5 Sv over 10 fractions. Mendes et al^8^ employed simulation techniques using the MCNPx code to calculate the risk of SC. They utilized a virtual phantom called the VW phantom, which represented a female with 63 different organs and limbs, a height of 165 cm, and a weight of 98 kg. A parallel field of 6 Mv was considered for treatment, and the MCNPX code calculated the absorbed dose in each organ. The risk of SC was then determined using the BEIR VII method. Overall, the methods for calculating the risk of SC encompass simulation, database analysis, mathematical calculations using proposed models, and more recently, ML models. This study investigated and contrasted different approaches to gain a thorough comprehension of the SC risk. Table 5 presents a comprehensive analysis of various techniques utilized in forecasting the likelihood of SC in the breast. The data presented in the table pertains to the occurrence of SC. It provides a comprehensive comparison of different methodologies, namely WBRT, APBI, SIB 2 volume, SIB 3 volume FP IMRT, SIB 3 volume IP IMRT, MCNPX, and our research findings, concerning the risk of SC. It also encompasses details regarding the methodology employed by various research groups, data sets utilized, follow‐up duration, age at exposure, average dose measured in Sv, dose range in Sv, cancer endpoint, and the SC risk. The patient's exposure to radiation differed among the different approaches, resulting in varying incidence ranges. This discrepancy highlights the variability in the risk of SC.

**TABLE 5:** Comparison of different methods for predicting the risk of SC in the breast, bladder, colon, esophagus, liver, lung, thyroid, and stomach.

Method	Name of groups	Cases/death	Age at exposure (years)	Follow up (years)	Average dose (Sv)	Dose range (Sv)	Cancer end point	SC risk %
Second cancer risk for leukemia								
WBRT	Donovan et al. (2012)^60^	566	35 to 80	0 to 5	0.60	0 to 40	Incidence	0.60
APBI	Donovan et al. (2012)^60^	182	35 to 80	0 to 5	0.19	0 to 38.5	Incidence	0.18
SIB 2 volume	Donovan et al. (2012)^60^	691	35 to 80	0 to 5	0.74	0 to 74	Incidence	0.69
SIB 3 volume FP IMRT	Donovan et al. (2012)^60^	605	35 to 80	0 to 5	0.43	0 to 36	Incidence	0.61
SIB 3 volume IP IMRT	Donovan et al. (2012)^60^	1094	35 to 80	0 to 5	1.17	0 to 53	Incidence	1.10
MCNPX	Mendes et al. (2017)^8^	548	35 to 80	0 to 5	0.27	0 to 40	Incidence	0.55
ML	Syleouni et al. (2023)^21^	200	62 to 85	0 to 5	Unknown	Unknown	Incidence	6.00
This Work ML	Sadeghi et al. (2024)	140	<30 to >75	5 to >40	0.31	0 to 0.98	Incidence	0.59
This Work ML	Sadeghi et al. (2024)	34	<1	0 to >52	0.69	0.01 to 7.1	Incidence	0.70
Second cancer risk for bladder								
WBRT	Donovan et al. (2012)^60^	8	35 to 80	0 to 5	0.04	0 to 40	Incidence	0.01
APBI	Donovan et al. (2012)^60^	8	35 to 80	0 to 5	0.04	0 to 38.5	Incidence	0.01
SIB 2 volume	Donovan et al. (2012)^60^	13	35 to 80	0 to 5	0.07	0 to 74	Incidence	0.01
SIB 3 volume FP IMRT	Donovan et al. (2012)^60^	13	35 to 80	0 to 5	0.05	0 to 53	Incidence	0.01
SIB 3 volume IP IMRT	Donovan et al. (2012)^60^	8	35 to 80	0 to 5	0.05	0 to 53	Incidence	0.01
MCNPX	Mendes et al. (2017)^8^	11	35 to 80	0 to 5	0.02	0 to 40	Incidence	0.01
This Work ML	Sadeghi et al. (2024)	15	<35 to >55	1 to 51	unknown	0 to 0.17	Incidence	0.36
This Work ML	Sadeghi et al. (2024)	142	<25 to >55	5 to >35	2.18	0 to >4.85	Incidence	0.39
Second cancer risk for colon								
WBRT	Donovan et al. (2012)^60^	65	35 to 80	0 to 5	0.08	0 to 40	Incidence	0.06
APBI	Donovan et al. (2012)^60^	50	35 to 80	0 to 5	0.07	0 to 38.5	Incidence	0.05
SIB 2 volume	Donovan et al. (2012)^60^	86	35 to 80	0 to 5	0.15	0 to 74	Incidence	0.09
SIB 3 volume FP IMRT	Donovan et al. (2012)^60^	137	35 to 80	0 to 5	0.21	0 to 53	Incidence	0.14
SIB 3 volume IP IMRT	Donovan et al. (2012)^60^	65	35 to 80	0 to 5	0.11	0 to 53	Incidence	0.06
MCNPX	Mendes et al.(2017)^8^	28	35 to 80	0 to 5	0.06	0 to 40	Incidence	0.03
This Work ML	Sadeghi et al. (2024)	73	13 to 88	0 to 60	1.2	<0.6 to 6.65	Incidence	0.38
This Work ML	Sadeghi et al. (2024)	47	23 to 65	5 to 49	3.2	<2.41 to >3.73	Incidence	0.41
This Work ML	Sadeghi et al. (2024)	226	<25 to >55	5 to >35	4.1	0 to >7.85	Incidence	0.41
Second cancer risk for esophagus								
WBRT	Donovan et al. (2012)^60^	27	35 to 80	0 to 5	0.12	0 to 40	Incidence	0.03
APBI	Donovan et al. (2012)^60^	21	35 to 80	0 to 5	0.11	0 to 38.5	Incidence	0.02
SIB 2 volume	Donovan et al. (2012)^60^	431	35 to 80	0 to 5	2.10	0 to 74	Incidence	0.43
SIB 3 volume FP IMRT	Donovan et al. (2012)^60^	82	35 to 80	0 to 5	0.37	0 to 53	Incidence	0.08
SIB 3 volume IP IMRT	Donovan et al. (2012)^60^	128	35 to 80	0 to 5	0.64	0 to 53	Incidence	0.13
This Work ML	Sadeghi et al. (2024)	148	<25 to >55	5 to >35	5.55	0 to >10.16	incidence	0.29
Second cancer risk for liver								
WBRT	Donovan et al. (2012)^60^	17	35 to 80	0 to 5	0.16	0 to 40	Incidence	0.02
APBI	Donovan et al. (2012)^60^	21	35 to 80	0 to 5	0.19	0 to 38.5	Incidence	0.02
SIB 2 volume	Donovan et al. (2012)^60^	29	35 to 80	0 to 5	0.29	0 to 74	Incidence	0.03
SIB 3 volume FP IMRT	Donovan et al. (2012)^60^	18	35 to 80	0 to 5	0.16	0 to 53	Incidence	0.02
SIB 3 volume IP IMRT	Donovan et al. (2012)^60^	28	35 to 80	0 to 5	0.26	0 to 53	incidence	0.03
MCNPX	Mendes et al. (2017)^8^	87	35 to 80	0 to 5	0.64	0 to 40	Incidence	0.09
WBRT	Donovan et al. (2012)^60^	116	35 to 80	0 to 5	0.68	0 to 40	Incidence	0.12
APBI	Donovan et al. (2012)^60^	74	35 to 80	0 to 5	0.7	0 to 38.5	Incidence	0.07
SIB 2 volume	Donovan et al. (2012)^60^	1108	35 to 80	0 to 5	1.9	0 to 74	Incidence	1.11
SIB 3 volume FP IMRT	Donovan et al. (2012)^60^	295	35 to 80	0 to 5	0.27	0 to 53	Incidence	0.30
SIB 3 volume IP IMRT	Donovan et al. (2012)^60^	675	35 to 80	0 to 5	1.8	0 to 53	Incidence	0.68
MCNPX	Mendes et al. (2017)^8^	384	35 to 80	0 to 5	0.22	0 to 40	Incidence	0.38
This Work ML	Sadeghi et al. (2024)	30	<45 to >55	1 to >10	7.2	0 to >21	Incidence	0.77
This Work ML	Sadeghi et al. (2024)	19	8 to 74	5 to 61	0.75	0 to 8.98	Incidence	0.59
This Work ML	Sadeghi et al. (2024)	69	<24 to >38	0 to 50	0.84	0 to >8	Incidence	0.44
Second cancer risk for thyroid								
WBRT	Donovan et al. (2012)^60^	28	35 to 80	0 to 5	0.12	0 to 40	Incidence	0.03
APBI	Donovan et al. (2012)^60^	25	35 to 80	0 to 5	0.08	0 to 38.5	Incidence	0.02
SIB 2 volume	Donovan et al. (2012)^60^	45	35 to 80	0 to 5	0.15	0 to 74	Incidence	0.05
SIB 3 volume FP IMRT	Donovan et al. (2012)^60^	36	35 to 80	0 to 5	0.11	0 to 53	Incidence	0.04
SIB 3 volume IP IMRT	Donovan et al. (2012)^60^	34	35 to 80	0 to 5	0.11	0 to 53	Incidence	0.03
MCNPX	Mendes et al. (2017)^8^	79	35 to 80	0 to 5	0.2	0 to 40	Incidence	0.08
This Work ML	Sadeghi et al. (2024)	43	29 to 78	5 to >40	0.11	0.01 to 0.24	Incidence	0.73
This Work ML	Sadeghi et al. (2024)	60	0 to 15	<15 to >30	0.09	0.04 to 0.5	Incidence	0.64
This Work ML	Sadeghi et al. (2024)	81	0.4 to 16	0 to 46	0.24	0 to 0.53	Incidence	0.73
This Work ML	Sadeghi et al. (2024)	50	1 to 75	5 to 39	0.8	0 to 29.2	Incidence	0.67
Second cancer risk for stomach								
WBRT	Donovan et al. (2012)^60^	294	35 to 80	0 to 5	0.72	0 to 40	Incidence	0.29
APBI	Donovan et al. (2012)^60^	105	35 to 80	0 to 5	0.23	0 to 38.5	Incidence	0.11
SIB 2 volume	Donovan et al. (2012)^60^	286	35 to 80	0 to 5	0.67	0 to 74	Incidence	0.29
SIB 3 volume FP IMRT	Donovan et al. (2012)^60^	256	35 to 80	0 to 5	0.64	0 to 53	Incidence	0.26
SIB 3 volume IP IMRT	Donovan et al. (2012)^60^	260	35 to 80	0 to 5	0.64	0 to 53	Incidence	0.26
MCNPX	Mendes et al. (2017)^8^	392	35 to 80	0 to 5	0.58	0 to 40	Incidence	0.39
This Work ML	Sadeghi et al. (2024)	61	<35 to >55	1 to 51	14.8	0 to 14.8	Incidence	0.34

To assess the risk of BC, we employed two datasets, specifically 140 (Boice et al^34^) and 34 (Hildreth et al^35^). Our analysis revealed that the BC risk was found to be 0.59% and 0.70% in the respective datasets. Notably, the ML method exhibited a higher SC Risk in comparison with alternative methods. The findings of our study align with previous research on predicting the SC Risk for the breast, thereby indicating its potential for accurately predicting the risk of SC. These results are consistent with other methods employed in this field. The incidence of bladder SC as a cancer endpoint is found to be 0.01% for the alternative methods, whereas our employed method yields a higher incidence of 0.36% and 0.39%. The risk of bladder SC varies depending on the specific methodology employed. In contrast to other approaches, our method demonstrates an elevated risk of bladder SC. The SC risk of the bladder is determined using the dataset of Griem et al^23^ and Weiss et al.^22^ Table 5 also presents a comparison of the risk of SC for colon cancer using different methods. Similar to previous tables, this table includes various factors such as the names of the research groups, age, dose range, and incidence of primary cancer. Among the methods, the SIB 3 volume FP IMRT method demonstrates an incidence SC risk of 0.14. The remaining results from the other methods indicate an incidence of SC risks below 0.1%. Our findings reveal the incidence of SC risks of 0.38%, 0.41%, and 0.41% for datasets consisting of 73 individuals (Inskip et al^24^), 47 individuals (Darby et al^25^), and 226 individuals (Weiss et al^22^), respectively. In the same way, our findings indicate that the Weiss et al.^22^ dataset demonstrates a 0.29% risk of esophagus SC for incidence. Moreover, the SIB 2 volume method outperforms other approaches in predicting the incidence mode of esophagus SC risk by yielding a higher value. The findings of the prediction for three types of SCs, namely lung, thyroid, and stomach, are presented in the last rows of the table. Specifically, the prediction for secondary lung cancer is based on data from three datasets: Van Leeuwen et al,^28^ Mattsson et al,^26^ and Davis et al.^29^ According to these sources, the prediction of the incidence of secondary lung cancer is 0.77%, 0.59%, and 0.44%. In comparison, our results are more closely aligned with the findings from SIB 3 volume IP IMRT and MCNPX calculations than with other methods. Notably, the results from SIB 2 Volume indicate a 1.11% SC risk, which is nearly twice as high as our estimate for the risk of lung SC. Studies involving underground miners have shown that a younger age at the time of exposure is associated with a heightened risk of developing lung cancer.^43^

Our study utilized data from four different research groups, namely Boice et al,^30^ Ron et al,^36^ Pottern et al,^37^ and Hall et al,^38^ to predict the occurrence of SC risk for the thyroid. These data encompassed various years and yielded for incidence the prediction of 0.73%, 0.64%, 0.73%, and 0.67%, respectively. In comparison with alternative approaches, our findings indicate higher predicted values. The predicted value of incidence for the SC stomach, based on the Griem et al^23^ dataset from Table 1, is 0.34%. Additionally, the results from various other methods for incidence are provided, and it is observed that most of these methods yield similar values. In individuals who have survived childhood cancer, the occurrence of primary hypothyroidism after radiation therapy is significantly elevated in females and in those who received treatment after the age of 15.^44^

Table 6 presents a comparison of the risk of SC in leukemia using two different methods: MCNPX and our ML methods. The first row of the table displays the results obtained through the MCNPX method, which identified 510 cases of leukemia. The age at exposure ranged from 35 to 80 years, the follow‐up duration was 0–5 years, and the average dose received was 0.79 Sv. The reported incidence of cancer was also provided, with an associated SC risk of 0.51%. Moving on to the second row, our ML method yielded results based on 163 cases of leukemia from Kaldor et al's^41^ study, as presented in Table 1. The age at exposure in this case was 42 years, the follow‐up duration ranged from 1 to 10 years, and the dose range was 0–30. The reported incidence of cancer was included as well. The third and fourth rows of the table present our findings from the ML method using data from Boivin et al^42^ and Damber et al^39^ studies, which consisted of 122 and 61 cases of leukemia, respectively. The reported incidence of cancer was also provided for these cases. It should be emphasized that the two different methods employed in this study may have utilized distinct assumptions and calculations, resulting in variations in the reported SC risk. Specifically, the MCNPX simulation predicted a 0.51% SC risk in leukemia, while our ML method yielded findings of 0.69%, 0.60%, and 0.70% when different datasets were utilized. Despite these variations, the results demonstrate agreement between the two methods used. To establish the most accurate and reliable approach for predicting SC risk in leukemia patients, further analysis and evaluation of these methods are necessary.

**TABLE 6:** A comparison of MCNPX and ML methods used to assess the risk of SC in leukemia patients.

Method	Name of groups	Cases/death	Age at exposure (years)	Follow up (years)	Average dose (Sv)	Dose range (Sv)	Cancer end point	SC risk %
Second cancer risk for leukemia								
MCNPX	Mendes et al. (2017)^8^	510	35 to 80	0 to 5	0.79	0 to 40	Incidence	0.51
This Work ML	Sadeghi et al. (2024)	163	42	1 to >10	Unknown	0 to >20	Incidence	0.69
This Work ML	Sadeghi et al. (2024)	122	<15 to >55	1 to 44	Unknown	0 to >30	Incidence	0.60
This Work ML	Sadeghi et al. (2024)	61	<20 to >70	0 to >19.6	0.39	<0.06 to >1.04	Incidence	0.70

An increased susceptibility to developing an SC was noted when compared to the overall populace. Furthermore, it was discovered that the risk of SC is particularly high among individuals who received a cancer diagnosis before reaching the age of 50 and those who have survived for at least 10 years. Cancer patients must undergo continuous monitoring not only for the recurrence of their initial cancer, but also for the development of new primary cancer. It is a frequent observation that both initial cancers and SCs often have similar risk factors related to lifestyle. This highlights the importance of promoting healthier lifestyles not only among the general population but also among individuals who have survived cancer. Standardized incidence ratios were calculated to assess the relative risks of SCs in individuals who have survived cancer, in comparison to the risks observed in the general population. The ratios were grouped into different categories, taking into account several factors such as the type of primary cancer, gender, age at the time of initial diagnosis, period of initial diagnosis, duration of survival, and location of the subsequent cancer. Table 7 presents the data from Neugut et al^61^ and Feller et al,^62^ indicating that various ML techniques may produce distinct risk estimates for SC risk in Hodgkins, larynx, multiple myeloma, pancreas, prostate, and rectum. The findings suggest that the choice of ML method can significantly influence the risk estimates for these specific types of cancer. The utilization of our ML approach resulted in predictions of 0.77%, 0.41%, 0.78%, 0.40%, 0.4%, and 0.31% for the datasets provided by Damber et al,^39^ Griem et al,^23^ Damber et al,^39^ Inskip et al,^24^ Weiss et al,^22^ and Darby et al,^25^ respectively.

**TABLE 7:** Comparison of secondary cancer (SC) risk for Hodgkins, Larynx, multiple myeloma, pancreas, prostate, and rectum with a population‐based study and maximum likelihood (ML) method.

Method	Name of groups	Cases/death	Age at exposure (years)	Follow‐up (years)	Average dose (Sv)	Dose range (Sv)	Cancer endpoint	SC risk %
Second cancer risk for Hodgkins								
A population‐based study	Neugut et al. (1995)^61^	2	Unknown	Minimum 6 months	Unknown	Unknown	Incidence	0.22
	Feller et al. (2020)^62^	67	Unknown	5 to 10	Unknown	Unknown	Incidence	1.71
This Work ML	Sadeghi et al. (2024)	17	<20 to >70	0 to >19.6	0.39	<0.06 to >1.04	Incidence	0.77
Second cancer risk for larynx								
A population‐based study	Feller et al. (2020)^62^	317	Unknown	5 to 10	Unknown	Unknown	Incidence	1.77
This Work ML	Sadeghi et al. (2024)	9	<35 to >55	1 to 51	0.08	0 to 0.08	Incidence	0.41
Second cancer risk for multiple myeloma								
A population‐based study	Feller et al.(2020)^62^	291	Unknown	5 to 10	Unknown	Unknown	Incidence	1.26
This Work ML	Sadeghi et al. (2024)	65	<20 to >70	0 to >19.6	0.39	<0.06 to >1.04	Incidence	0.78
Second cancer risk for pancreas								
A population‐based study	Feller et al.(2020)^62^	669	Unknown	5 to 10	Unknown	Unknown	Incidence	1.36
This Work ML	Sadeghi et al. (2024)	35	13 to 88	0 to 60	0.16	<0.08 to 0.87	Incidence	0.40
Second cancer risk for prostate								
A population‐based study	Neugut et al. (1995)^61^	292	Unknown	Minimum 6 months	Unknown	Unknown	Incidence	1.18
	Feller et al. (2020)^62^	3694	Unknown	5 to 10	Unknown	Unknown	Incidence	0.67
This Work ML	Sadeghi et al. (2024)	176	<25 to >55	5 to >35	2.18	0 to >4.85	Incidence	0.41
Second cancer risk for rectum								
A population‐based study	Neugut et al. (1995)^61^	139	Unknown	Minimum 6 months	Unknown	Unknown	Incidence	0.86
	Feller et al. (2020)^62^	2430	Unknown	5 to 10	Unknown	Unknown	Incidence	0.18
This Work ML	Sadeghi et al. (2024)	14	23 to 65	5 to 49	4.9	<3.32 to >6.51	Incidence	0.31

The findings presented in Table 8 solely provide information on the risk of SC in different areas of the body, specifically in the bone, NMSC, and NHL as determined by our methodology. It highlights the necessity for novel modeling techniques to accurately forecast the probability of developing SC after undergoing RT. Various factors contribute to the likelihood of SC, encompassing the radiation dose and volume administered to the patient, the patient's age during RT, the specific organ or tissue subjected to irradiation, and genetic predisposition. The reference provided by Boice et al^30^ served as the source for the dataset utilized in this research, consisting of 15 data points. Our results indicate a SC risk of 0.80% in the SC bone. Furthermore, the total exposure of red bone marrow to diagnostic radiation varies with the calendar year, showing notable increases around 1950 and after 1980, with men typically experiencing greater doses compared with women.^45^

**TABLE 8:** Outcomes of the risk of secondary cancer (SC) in the bone, non‐melanoma skin cancer (NMSC), and non‐Hodgkin lymphoma (NHL) using our maximum likelihood (ML) method.

Method	Name of groups	Cases/death	Age at exposure (years)	Follow‐up (years)	Average dose (Sv)	Dose range (Sv)	Cancer endpoint	SC risk %
Secondary cancer risk for bone								
This Work ML	Sadeghi et al. (2024)	15	<30 to >75	1 to >40	22	0 to >32	Incidence	0.80
Second cancer risk for NMSC								
This Work ML	Sadeghi et al. (2024)	41	1 to 19	10 to 49	5	0 to 5.5	Incidence	0.66
This Work ML	Sadeghi et al. (2024)	24	<1	5 to >48	2.25	0 to >2.25	Incidence	0.68
This Work ML	Sadeghi et al. (2024)	14	<19 to >40	0 to 45	2.6	0 to >2.6	Incidence	0.70
Second cancer risk For NHL								
This Work ML	Sadeghi et al. (2024)	81	<20 to >70	0 to >19.6	0.39	<0.06 to >1.04	Incidence	0.76
This Work ML	Sadeghi et al. (2024)	16	0 to 15	0 to >28	0.3	0 to >0.3	Incidence	0.50
This Work ML	Sadeghi et al. (2024)	21	<35 to >55	1 to 51	1.55	0 to 1.55	Incidence	0.31

Nevertheless, despite notable advancements in mathematical and theoretical modeling, no single reference model has successfully encompassed all the parameters due to the intricate non‐linear relationships between them. Additionally, the existing models have failed to take into account the influence of factors such as genetics, hormones, lifestyle, radiation from secondary particles in RT, and the dose received from RT. This underscores the imperative need for novel modeling techniques that incorporate all relevant factors, including those previously unconsidered, to accurately predict the likelihood of SC after RT. The application of our ML approach yielded predictions of NMSC SC risk at 0.66% (incidence), 0.68% (incidence), and 0.70% (incidence) for the datasets obtained from Shore et al,^31^ Hildreth et al,^32^ and Shore et al^33^ respectively. Additionally, the NHL SC risk was estimated to be 0.76% (incidence), 0.50% (incidence), and 0.31% (incidence) for the datasets provided by Damber et al,^39^ Ron et al,^40^ and Griem et al,^23^ respectively.

## 4 SUMMARY AND CONCLUSION

This investigation primarily aims to enhance the understanding and prediction of SC risk that may arise as a result of RT. The risk of developing SC in various types of cancer, such as primary pancreas, breast, colon, bladder, esophagus, lung, thyroid, stomach, and leukemia cancer, has been assessed using different methods. The research compares various methods, including simulation, database analysis, mathematical calculations, and ML models, to offer an extensive knowledge of the risk of SC. The findings reveal discrepancies in the reported SC risk, which are contingent upon the particular methodology employed. This underscores the necessity for additional analysis and evaluation to ascertain the most precise approach. The paper underscores the necessity for novel modeling techniques that can effectively mitigate the SC risk. To achieve this, ML models are employed to establish a practical framework for forecasting the occurrence of SC using patient data. However, existing models have certain limitations, such as their inability to capture all relevant parameters. To address these limitations, the paper employs a dataset comprising instances and attributes to educate and assess ML models for predicting the occurrence of SC based on patient data. It not only enhances our understanding and prediction of SC following RT but also facilitates the development of personalized treatment approaches. Furthermore, it allows for the incorporation of patient data into ML models, thereby improving their effectiveness and applicability in clinical settings. Implementing the proposed automated model in population‐wide screening programs can assist physicians in identifying SCs in asymptomatic individuals, potentially reducing cancer‐related mortality. Identifying SC at an early stage can significantly enhance a patient's chance of survival by allowing for timely and appropriate treatment. By accurately detecting SC, healthcare providers can tailor treatment plans more effectively, potentially improving the efficacy of therapies and reducing unnecessary treatments. Additional research is required to obtain a comprehensive understanding of the particular forms of cancer that are predominantly impacted by the selection of ML techniques. This is crucial to enhance the precision of risk assessments and advance the accuracy of cancer predictions.

## AUTHOR CONTRIBUTIONS

**Hossein Sadeghi:** Conceptualization (equal); methodology (equal); project administration (equal); software (equal); supervision (equal); validation (equal); writing – original draft (equal). **Fatemeh Seif:** Conceptualization (equal); data curation (equal); funding acquisition (equal); methodology (equal); project administration (equal); supervision (equal); writing – original draft (equal). **Erfan Hatamabadi Farahani:** Formal analysis (equal); methodology (equal); software (equal); writing – original draft (equal). **Soraya Khanmohammadi:** Conceptualization (equal); investigation (equal); methodology (equal); software (equal); validation (equal); visualization (equal); writing – original draft (equal). **Shahla Nahidinezhad:** Data curation (equal); methodology (equal); resources (equal); validation (equal).

## FUNDING INFORMATION

The authors received no financial support for the research, authorship, and publication of this article.

## CONFLICT OF INTEREST STATEMENT

The authors declare no potential conflict of interest.

## Data Availability

All data supporting the findings of this study are available within the paper. Source code and Datasets are available from: https://github.com/Sorayakhan/khanmohammadi/blob/main/Hatam‐FINAL‐Paper‐GitHub.ipynb.
